# ID1 and ID4 Are Biomarkers of Tumor Aggressiveness and Poor Outcome in Immunophenotypes of Breast Cancer

**DOI:** 10.3390/cancers13030492

**Published:** 2021-01-27

**Authors:** Marta Garcia-Escolano, Yoel G. Montoyo-Pujol, Fernando Ortiz-Martinez, Jose J. Ponce, Silvia Delgado-Garcia, Tina A. Martin, Hortensia Ballester, F. Ignacio Aranda, Elena Castellon-Molla, J. Miguel Sempere-Ortells, Gloria Peiro

**Affiliations:** 1 Research Department, University General Hospital of Alicante, and Alicante Institute for Health and Biomedical Research (ISABIAL), Pintor Baeza 12, 03010 Alicante, Spain; montoyo_yoe@gva.es (Y.G.M.-P.); fernando.ortiz@qiagen.com (F.O.-M.); peiro_glo@gva.es (G.P.); 2 Medical Oncology Department, University General Hospital of Alicante, and Alicante Institute for Health and Biomedical Research (ISABIAL), Pintor Baeza 12, 03010 Alicante, Spain; ponce_joslor@gva.es; 3 Gynecology and Obstetrics Department, University General Hospital of Alicante, and Alicante Institute for Health and Biomedical Research (ISABIAL), Pintor Baeza 12, 03010 Alicante, Spain; delgado_sil@gva.es (S.D.-G.); martin_tin@gva.es (T.A.M.); ballester_hor@gva.es (H.B.); 4 Pathology Department, University General Hospital of Alicante, and Alicante Institute for Health and Biomedical Research (ISABIAL), Pintor Baeza 12, 03010 Alicante, Spain; aranda_ign@gva.es (F.I.A.); castellon_ele@gva.es (E.C.-M.); 5 Biotechnology Department, Immunology Division, University of Alicante, Ctra San Vicente s/n. 03080-San Vicente del Raspeig, 03010 Alicante, Spain; josemiguel@ua.es

**Keywords:** inhibitor of differentiation (ID), mRNA expression, breast carcinoma, immunophenotype, prognosis

## Abstract

**Simple Summary:**

Inhibitor of differentiation (ID) proteins are essential to promote proliferation during embryonic development, but they are silenced in most adult tissues. Evidence to date shows ID1 expression in many tumor types, including breast cancer. However, the role of the remaining ID family members, especially ID4, in breast cancer remains unclear. In this work, we aimed to assess the four ID genes expression in breast cancer cell lines and a long series of breast cancer samples and correlate them with clinicopathological features and patients’ survival. We observed a significantly higher expression of *ID4* in tumor cell lines than the healthy breast epithelium cell line. We confirmed that the overexpression of *ID1* and *ID4* correlated with more aggressive phenotypes and poor survival in breast cancer patients’ samples. Our results support the importance of ID proteins as targets for the development of anti-cancer drugs.

**Abstract:**

Inhibitor of differentiation (ID) proteins are a family of transcription factors that contribute to maintaining proliferation during embryogenesis as they avoid cell differentiation. Afterward, their expression is mainly silenced, but their reactivation and contribution to tumor development have been suggested. In breast cancer (BC), the overexpression of ID1 has been previously described. However, whether the remaining *ID* genes have a specific role in this neoplasia is still unclear. We studied the mRNA expression of all ID genes by q RT-PCR in BC cell lines and 307 breast carcinomas, including all BC subtypes. Our results showed that ID genes are highly expressed in all cell lines tested. However, ID4 presented higher expression in BC cell lines compared to a healthy breast epithelium cell line. In accordance, ID1 and ID4 were predominantly overexpressed in Triple-Negative and HER2-enriched samples. Moreover, high levels of both genes were associated with larger tumor size, histological grade 3, necrosis and vascular invasion, and poorer patients’ outcomes. In conclusion, ID1 and ID4 may act as biomarkers of tumor aggressiveness and worse prognosis in breast cancer, and they could be used as potential targets for new treatments discover.

## 1. Introduction

Breast cancer (BC) is the most common cancer diagnosed among women and is still the leading cause of cancer-related death in this gender worldwide [1]. In the last decades, we have witnessed the appearance of new diagnosis methods that have increased the diagnosis of early stages of BC [2]. This fact, added to the implementation of novel therapies [3], has improved BC patients’ prognosis. Indeed, the current 5-year survival rate is 91.1% [4]. Despite these advances, about 30% of BC patients relapse [5]. Additionally, the 5-year survival rate decreases dramatically to 27% for patients diagnosed with advanced stages of the disease [6], and there are still no specific treatments for the triple-negative breast cancer (TNBC) subtype [7]. These data bring to light the need for finding new targets for the efficient treatment of the most aggressive breast tumors. 

The inhibitor of differentiation (ID) proteins are a family of four (ID1-4) helix-loop-helix (HLH) transcription factors that lack the DNA binding domain. They act by recruiting other transcription regulators forming homo- or hetero-dimers that fail to bind the E-sequences of DNA. As a result, the transcription of E-box dependent genes is inhibited [8]. IDs sequence and structure are very conserved among family members and species, especially the HLH domain, indicating that they exert an important function [9,10]. In fact, ID proteins play a crucial role during development, where they regulate cell fate by impairing senescence [11]. Despite this, the ID expression patterns usually overlap, and it is known that they are differentially regulated, which suggests different functions for each of them. In adulthood, their expression is mainly restricted to some populations of stem cells [12,13,14] or tissues that proliferate as an injury response [15]. 

The activation and participation of the ID family, especially ID1, in tumor development have been widely studied in several tumor types. For instance, the expression of ID1 has generally been associated with malignant behavior and poorer prognosis in the stomach, colon, prostate, ovary, bladder, pancreas, and brain tumors, among others (reviewed in [16]). In contrast, in other cancer types such as hepatic or thyroid tumors, the role of ID1 is not so evident [17,18,19,20]. In BC, most of the studies carried out included a short clinical series or experimental cell models. Data reported have associated ID1 with hormone receptors (HR) negative status, aggressive phenotypes, and shorter survival [21,22,23,24]. Nevertheless, some authors have obtained different results [25].

In contrast to ID1, little data are available about the contribution of the other ID proteins to tumor malignancy. Previous investigators have demonstrated an association of ID2 expression with tumor development, aggressiveness, and chemotherapy resistance [26,27,28]. However, in BC, there is also evidence supporting the opposite role [29]. Similarly, recent studies postulate that ID3 expression may contribute to chemo-resistance [30,31], while others claim that it may reverse multi-drug resistance and increase chemosensitivity [32,33].

It is interesting to highlight the controversy surrounding ID4. It is the longest protein of this family [34], and it shows expression patterns that differ from the other three family members [10,35]. In fact, ID4 has been proposed as a regulatory factor of the expression of the remaining ID proteins [36]. Increased expression of ID4 has been observed in some tumor types such as hepatocellular carcinoma [37], glioblastoma [38], or melanoma [39], indicating a possible role in tumor development. On the contrary, other authors have reported that loss of ID4 by hypermethylation increases tumor progression and metastasis [40,41], supporting the theory of *ID4* being a tumor suppressor gene. In BC, the interest in ID4 has generally been focused on another direction. It is known that, in the breast, there is a negative regulation loop between ID4 and BRCA1 [42], and most of the studies published to date investigate the dysregulation of this loop during BC development [43]. 

Here, we aimed to assess the contribution of *ID* genes in BC malignancy and clarify the dual role of *ID4* in this neoplasia. To that end, we analyzed the expression of the four *ID* genes in BC cell lines and a large clinical series of BC patients, previously stratified by immunophenotypes. mRNA expression results were correlated with clinicopathological parameters and patients’ outcome. We found that *ID4* presented high mRNA expression in BC cell lines. Moreover, in the analyzed clinical series, its overexpression, together with *ID1*, was associated with aggressive clinic-pathological factors and poor survival. 

## 2. Results

### 2.1. ID Genes Expression in BC Cell Lines

**Figure 1:**
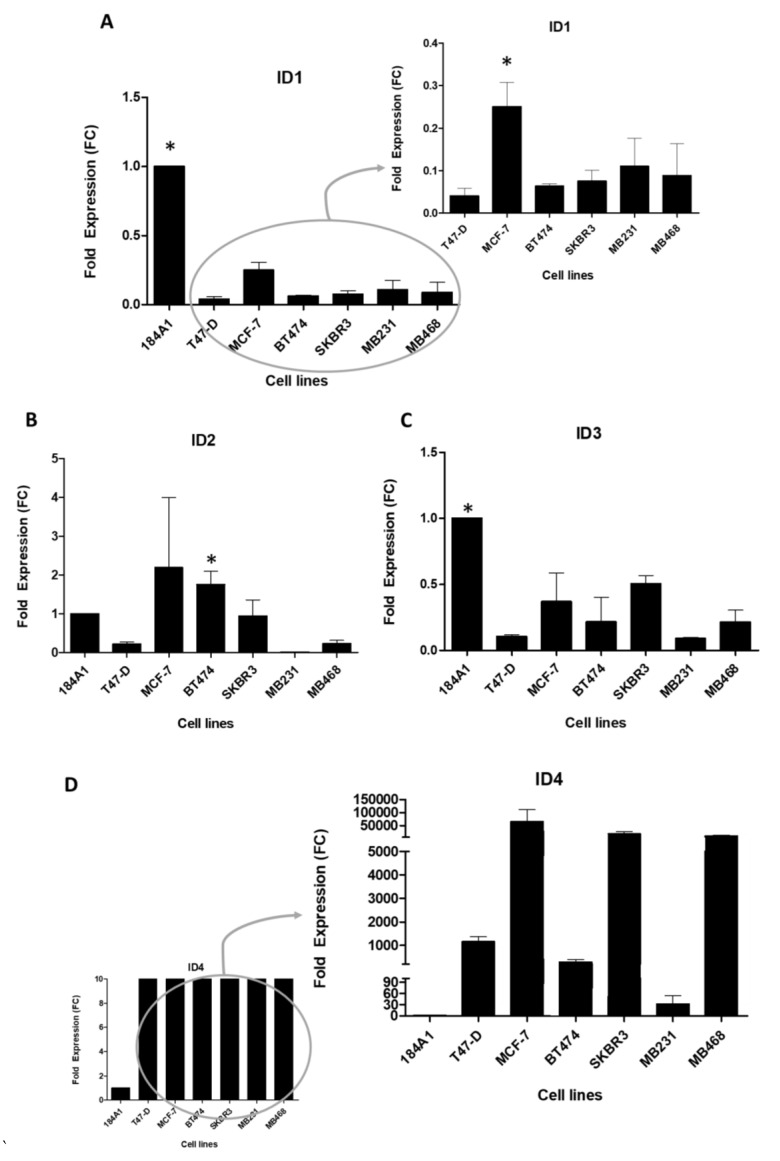
mRNA expression of ID genes (**A**: *ID1*, **B**: *ID2*; **C**: *ID3*; **D**: *ID4*) in six breast tumor cell lines (MCF-7, T47-D, BT-474, SK-BR-3, MDA-MB-231, and MDA-MB-468) and the breast epithelium cell line 184A1. All experiments were done three times. Each graph shows the average and standard derivation (error bars) of the three measurements. * *p* < 0.05 compared to all the other cell lines.

We detected high mRNA expression of the four *ID* genes in all cell lines, including the non-tumor cell line 184A1, which indeed showed significantly higher expression of *ID1* and *ID3* than all the tumor cell lines (all *p* ≤ 0.03). When we excluded 184A1 from the analysis, MCF-7 showed the highest levels of *ID1* compared with the other cell lines (all *p* ≤ 0.03). In contrast, *ID3* levels did not differ significantly among tumor cell lines. All cell lines showed a similar expression of *ID2*, except MDA-MB-231, which showed no expression. Regarding *ID4*, all tumor cell lines showed increased expression compared with 184A1 (all *p* ≤ 0.06), meaning that this gene could have a specific role in BC pathogenesis. Among the tumor cell lines, MCF-7 presented the highest levels of *ID4* expression (Figure 1).

### 2.2. Patients’ and Tumor Characteristics

In our clinical series of 307 non-consecutive patients with breast carcinoma, the mean age at diagnosis was 55.3 years (range 23–88). Tumors were predominantly larger than 20 mm (54.9%), with histological grade 3 (59.9%), absence of necrosis (65.1%) or vascular invasion (63.3%), and negative lymph nodes (61.2%). We classified samples into five molecular subtypes according to a validated IHC surrogate profile panel [44] as follows: 19.9% luminal A-like, 19.2% luminal B/HER2-negative, 15.6% luminal B/HER2-positive, 13.4% HER2-enriched, and 31.9% TNBC. All patients had a follow-up (FU) at least 12 months, being the median FU 62 months (P25 = 40 months; P75 =113 months). The median OS was 62 months (range 2–295 months), whereas the median period to local recurrence or distant metastasis was 45 months (range 2–214 months). 

### 2.3. ID Genes Are Overexpressed in Most Analyzed Tumor Samples

**Figure 2:**
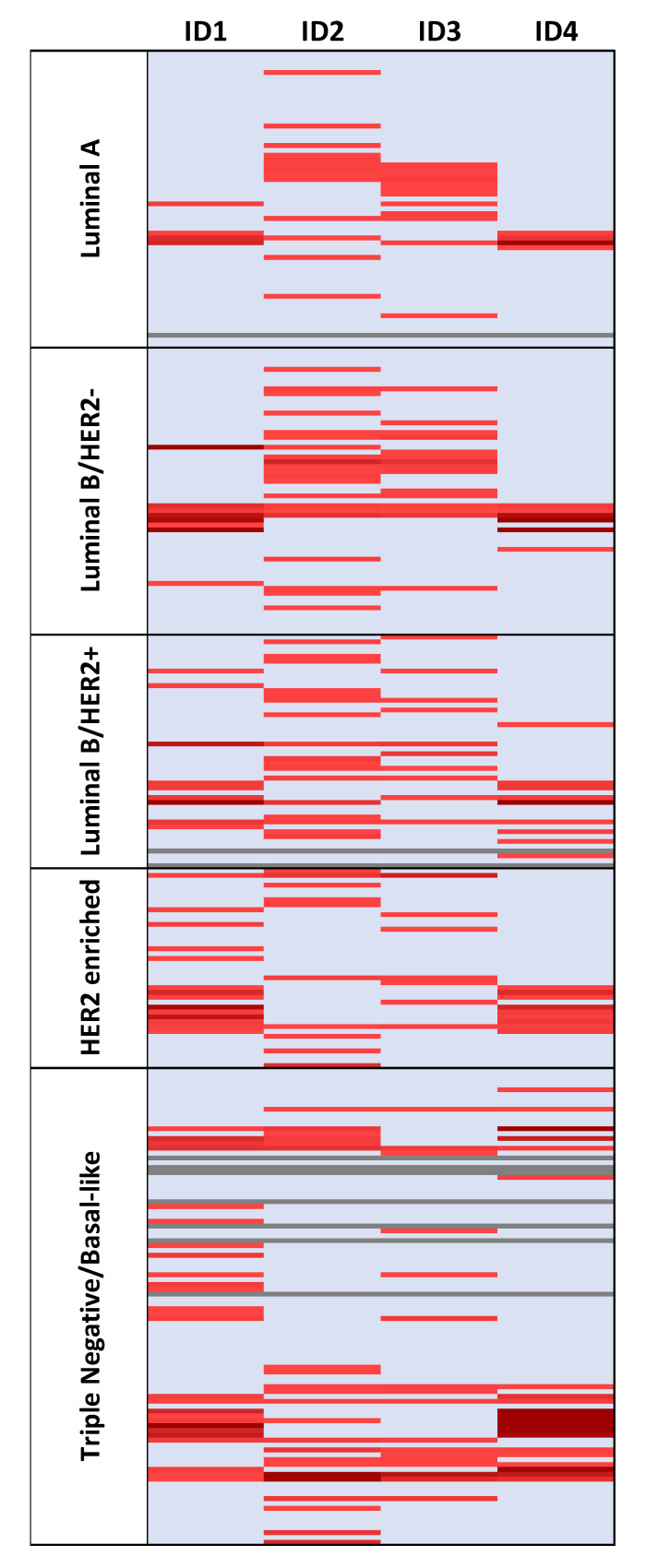
Heat map representing the expression of ID genes in all BC samples (*n* = 307) according to BC phenotypes. Normal–low expression (FC < 2) is represented in blue and overexpression (FC ≥ 2) is represented in red. Samples with no available data (*n* = 10) are colored gray.

As for the study exclusion criteria, we did not analyze *ID* genes’ expression in 10 samples. The remaining 297 samples were classified according to the expression of *ID* genes into two groups: overexpression (FC ≥ 2) and normal-low expression (FC < 2). Based on this, *ID1* was overexpressed in 61 samples (20.5%), *ID2* in 83 (27.9%), *ID3* in 61 (20.5%), and *ID4* in 49 (16.5%). When considering the *ID* family together, 50.5% of the studied tumors presented overexpression of at least one *ID* gene (Figure 2). Regarding these samples, 52.0% had only one *ID* gene overexpressed, whereas overexpression of the four *ID* genes simultaneously was found in 6.0% of them. 

### 2.4. ID1 and ID4 Overexpression Is Associated with Aggressive Clinicopathological Features

We found *ID1* and *ID4* overexpression in tumors larger than 20 mm (68.3%, *p* = 0.012; and 67.3%, *p* = 0.036, respectively), with histological grade 3 (78.7%, *p* = 0.029; and 79.6%, *p* = 0.042, respectively), and necrosis (50.0% *p* = 0.003; and 52.1% *p* = 0.004, respectively). Additionally, the overexpression of *ID4* was associated with the presence of vascular invasion (51.0%, *p* = 0.015) but as a trend for *ID1* overexpression (45.9%, *p* = 0.055). Despite most of the samples with high expression of *ID4* were from patients older than 50 (73.5%), this association was marginally significant (*p* = 0.071). All data shown in Table 1.

A trend towards significance was observed between *ID2* overexpression and necrosis (72.3%, *p* = 0.091). No significant associations were found for *ID3* (Table S1).

**Figure 3:**
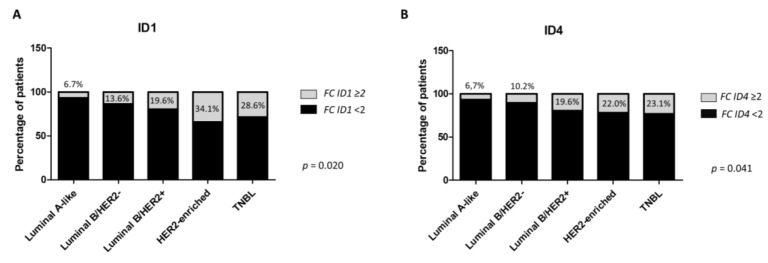
Percentage of samples overexpressing *ID1* (**A**) and *ID4* (**B**) at mRNA level in each breast cancer immunophenotype. TNBL, triple-negative basal-like.

Regarding the intrinsic BC subtypes, *ID1* and *ID4* were predominantly overexpressed in the aggressive subtypes TNBC and HER2-enriched (*p* = 0.020 and *p* = 0.041, respectively) (Figure 3). In contrast, *ID2* and *ID3* overexpression were more frequently found in luminal B subtypes, although differences among phenotypes were not significant (*p* > 0.05) (Figure S1).

### 2.5. Patients Expressing High Levels of ID1 and ID4 Present Shorter OS and DFS

Univariate analyses (Table 2) revealed that patients with larger tumor size (HR: 2.8; 95% CI = 1.5, 5.2; *p* = 0.002), histological grade 3 (HR: 8.1; 95% CI = 1.1, 58.8; *p* = 0.040), necrosis (HR: 1.8; 95% CI = 1.0, 3.2; *p* = 0.042), vascular invasion (HR: 2.6; 95% CI = 1.4, 4.6; *p* = 0.001), positive lymph-nodes (HR: 2.6; 95% CI = 1.4, 4.6; *p* = 0.001), absence of hormone receptors (HR: 1.8; 95% CI = 1.0, 3.3; *p* = 0.039) and *ID4* overexpression (HR: 2.1; 95% CI = 1.2, 3.9; *p* = 0.016) had shorter OS. 

Similarly, DFS was significantly affected by larger tumor size (HR: 1.8; 95% CI = 1.0, 3.2; *p* = 0.034), presence of necrosis (HR: 1.8; 95% CI = 1.0, 3.1; *p* = 0.004), vascular invasion (HR: 2.5; 95% CI = 1.4, 4.2; *p* = 0.001), *ID1* (HR: 2.0; 95% CI = 1.1, 3.6; *p* = 0.016), and *ID4* overexpression (HR: 3.4; 95% CI = 1.9, 6.0; *p* <0.001). A trend was also observed for HR negative status (HR: 1.6; 95% CI = 0.9, 2.8; *p* = 0.080) (Table 2). 

**Figure 4:**
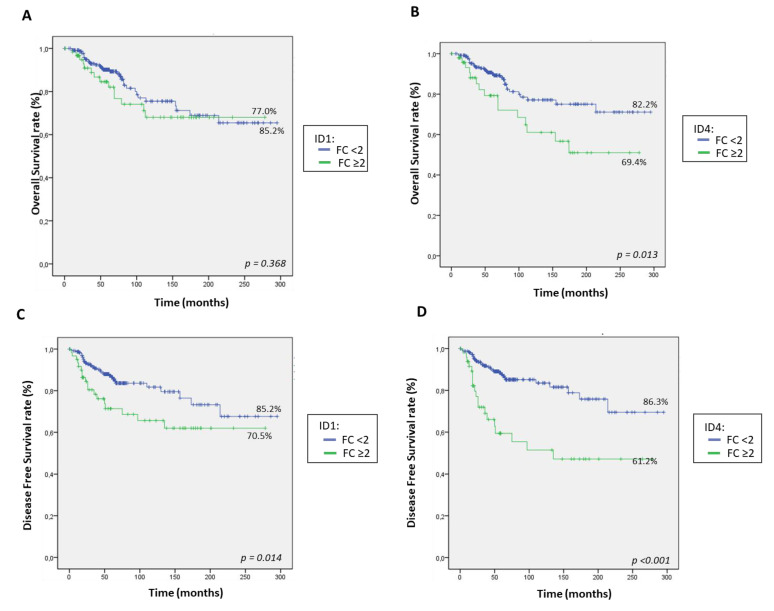
Kaplan–Meier plot for overall survival (**A**,**B**) and disease-free survival (**C**,**D**) rates (%) for all patients classified according to tumor *ID1* (**A**,**C**) or *ID4* (**B**,**D**) mRNA expression.

We performed Kaplan–Meier curves for OS and DFS (Figure 4) and observed that patients whose tumors had high levels of *ID1* (FC ≥ 2) showed shorter OS (77.0% vs. 85.2%) and DFS (70.5% vs. 85.2%) rates. However, these differences were only significant for DFS (*p* = 0.014). Likewise, tumors with *ID4* overexpression presented significantly decreased rates of OS (69.4% vs. 82.2%; *p* = 0.013) and DFS (61.2% vs. 86.3%; *p* < 0.001). Neither *ID2* nor *ID3* overexpression showed significant associations with OS or DFS). 

Patients were further stratified according to BC intrinsic immunophenotypes, and Kaplan–Meier curves for OS and DFS were carried out (Tables S2 and S3, and Figure S2). Results showed that patients with HER2-enriched tumors had the poorest OS rates (65.9%, *p* = 0.027) and DFS rates (58.5%, *p* < 0.001). Focusing specifically on this subgroup of patients, those whose tumors expressed high levels (FC ≥ 2) of *ID1* or *ID4* presented markedly shorter OS and DFS rates (all *p* < 0.05) (Figure S3). On the contrary, neither *ID2* nor *ID3* overexpression was associated with a worse prognosis in this subgroup of BC patients. 

### 2.6. ID4 Is an Independent Marker of Poor Prognosis in Breast Cancer

All variables that significantly affected OS and DFS were included in Cox’s proportional hazard multivariate analysis (Table 2). Results showed that none of the chosen factors independently affected OS (all *p* > 0.05), although a trend towards significance was observed for *ID4* overexpression (*p* = 0.176). In contrast, the presence of vascular invasion (*p* = 0.031), necrosis (*p* = 0.050), and *ID4* overexpression (*p* = 0.001) were independent prognostic factors for DFS. 

### 2.7. Comparison with Available Databases 

We compared our results with those available in the public database Kaplan Meier Plotter [45]. Two different cohorts of BC patients were used for these analyses: DFS (*n* = 3951) and OS (*n* = 1402). In agreement with our series, the expression of *ID2* and *ID3* did not impact either in DFS or in OS, whereas higher expression of *ID1* and *ID4* was associated with poorer DFS (all *p* < 0.05). Nevertheless, contrary to our data, *ID4* did not significantly correlate with OS for these patients (Figure 4). 

## 3. Discussion

In the current study, we analyzed the presence of the four ID genes in BC and found that *ID4* mRNA expression was higher in BC cell lines than the healthy epithelium control cells. The expression of the remaining *ID* genes was also detected in all cell lines studied, although the expression in BC lines was not significantly higher than in the control model. In the light of these results, we took the investigation to the next step and studied the expression of the *ID* family in 307 non-consecutive samples of human BC stratified by immunophenotypes. As far as we know, our work represents the largest series where the mRNA expression of the four *ID* genes has been simultaneously studied to date. 

Our data showed that at least one of these genes was overexpressed in most analyzed tumor samples compared to healthy breast tissue, supporting their reactivation during breast carcinogenesis. More concretely, in our series, overexpression of *ID1* and *ID4* was related to HR-negative immunophenotypes, in contrast to *ID2* and *ID3,* which were more frequently overexpressed in luminal subtypes. This suggests that each gene could contribute to the etiopathogenesis of a specific subtype of BC. We further demonstrated that mRNA overexpression of *ID1* and *ID4* was linked to aggressive clinicopathological factors and poorer survival rates. Besides, *ID4* showed an independent prognostic value for recurrence. Our results regarding *ID1* are supported by previous studies demonstrating its expression in HR-negative BC phenotypes [22,46] and implied more aggressive features and poorer outcomes [22,23,24,46]. 

According to our knowledge, the contribution of *ID4* in BC aggressiveness is still unclear. As stated before, in the last decades, the role of *ID4* as a tumor suppressor gene has been proposed based on evidence showing an increase in tumor progression and the risk of metastasis following the loss of *ID4* by hypermethylation [40,41]. Nevertheless, opposite data claiming an increased expression of ID4 in various tumor types have also been reported [37,38,39]. Along with this work, we have presented compelling evidence that supports a pro-oncogenic role of this gene in BC progression. Our in vitro data demonstrates that the expression of *ID4* is higher in tumor cell lines than in healthy breast epithelium.

Moreover, our study in a large series of BC patients establishes an association between *ID4* overexpression and aggressive clinic-pathological features and BC subtypes. Additionally, *ID4* showed an independent prognostic value for recurrence. Comparably, other authors have studied ID4 expression at the protein level in BC specimens and found correlations with high tumor grade, absence of HR, increased vascularization and recurrence, and shorter OS [47,48,49,50,51,52]. The expression of ID4 has also been linked to a stem-like phenotype in the aggressive basal-like BC subtype [53]. In agreement, our work demonstrated that these associations are also found at the mRNA level. These data together support the involvement of *ID4* in the pathogenesis of BC beyond the dysregulation of BRCA1 expression. A very recent work carried out on a short series of basal-like BC samples and cell lines showed that the interaction between ID4 and DNA damage repair proteins could contribute to the DNA damage repair deficiencies and the aggressiveness of this BC subtype [54]. These data support the high association between *ID4* overexpression and the most aggressive BC phenotypes in our series.

Unfortunately, the current work did not contribute to elucidating the role of *ID2* and *ID3* in BC. Previous reports aimed at studying both genes in BC, and their conclusions were divergent [24,26,29]. Considering the above data and our results, it seems that these two genes have a secondary role in BC progression. Nevertheless, further research could be carried out to elucidate these discrepancies.

Interestingly, other authors have found opposite conclusions about *ID1* and *ID4* mRNA expression in BC. Zhou and colleagues recently evaluated the prognostic relevance of the ID family by using a set of public databases and reported that higher mRNA levels of *ID1* and *ID4* were associated with lower histological grades, less lymph-node metastasis, and longer survival rates [25]. As our results differ from those presented by Zhou et al. [25], we carried out a prognosis analysis with the data available at the database Kaplan–Meier Plotter. In agreement with our results, Kaplan–Meier Plotter data showed that high mRNA levels of *ID1* and *ID4* correlated with shorter DFS rates. In contrast, the overexpression of *ID2* or *ID3* showed no association with survival. 

The discordances found between *ID1* expression in cell lines and patient samples could be explained considering the nature of this gene. It is known that *ID1* acts as a driver of cell proliferation, and all the cell lines in our in vitro study are highly proliferative. Consequently, all studied cell lines, including the non-tumor cell line 184A1, presented high rates of *ID1* expression. This fact has previously been reported [55] and suggests that interactions between tumor and stromal cells may regulate *IDs* expression [56], which is an interesting field for further research, especially in the different subtypes of BC. Other in vitro studies have demonstrated that induced expression of ID1 transformed non-aggressive BC cell lines into more aggressive cells [21,26]. That is in agreement with our data since the less aggressive luminal cell lines, T47-D, showed reduced levels of ID1 compared to the invasive TNBC MDA-MB-231 and MDA-MB-468. 

In conclusion, our results support that *ID1* and *ID4* have a role as pro-oncogenes in BC and are associated with tumor aggressiveness in BC subtypes. In addition, they seem relevant molecular prognostic markers in this neoplasia. Our findings of *ID4* are especially interesting, considering the increasing controversy about the role of this gene in BC. Therefore, both genes seem potential targets for developing novel drugs for BC treatment, specifically in more aggressive phenotypes such as TNBC and HER2-enriched. Of note, this strategy is now more realistic considering that Wojnarowicz and colleagues have recently developed a molecule that successfully targets and disrupts ID proteins in vitro and in vivo [57]. Nevertheless, our results, which have potential therapeutic implications, warrant further investigation. 

## 4. Materials and Methods 

### 4.1. Cell Culture

Six human BC cell lines: MCF-7 and T47-D (luminal A-like), BT474 (luminal B/HER2-negative), SKBR3 (HER2-enriched), MDA-MB-231, and MDA-MD-468 (TNBC), and the normal breast epithelium cell line (184A1) were purchased from ATCC (Manassas, VA, USA). All tumor cell lines were maintained in 75 cm^2^ flask (SPL Life Science, Gyeonggi, Korea) fed with Dulbecco Modified Eagle Medium/Ham F12 (1:1) with L-glutamine and 15 mM HEPES media (Biowest, Nauaillé, France) supplemented with 10% Fetal Bovine Serum (Biowest, Nauaillé, France), 50 U/mL penicillin and 50 mg/mL streptomycin (Biowest, Nauaillé, France). The cell line 184A1 was maintained in Mammary Epithelial Cell Growth Medium (MEGM Bullekit^TM^ Lonza, Basel, Switzerland) supplemented with growth factors (MEGMTM SingleQuots^TM^ Supplements, Lonza, Basel, Switzerland), and 1% penicillin and streptomycin (Biowest, Nauaillé, France). Cells were incubated in humidified incubators at 37 °C and 5% CO_2_. 

Once a year, or when cells were thawed out, a mycoplasma test was carried out following the instructions of the Venor^®^GeM One Step kit (Minerva BioLabs, Berlin, Germany). Cells were maintained in culture for a few weeks, enough time for a couple of passages, and needed experiments.

### 4.2. Patients

Our retrospective study included 307 non-consecutive samples from patients with primary BC diagnosed and treated at the University General Hospital of Alicante (Spain) between 1993 and 2016. Tumor specimens were obtained from the Biobank collections of the same institution. Inclusion criteria were: diagnosis of resectable infiltrative primary breast carcinoma of at least 10 mm in size, stage I-III, complete pathological report, and those patients who had not been lost to follow-up in at least 12 months. Patients with tumors in stage IV, with preoperative chemo-/radiotherapy or low quality/quantity of RNA, were excluded from the study. The Local Ethics Committee approval was obtained for this project (ethic code PI2014/39), informed consent from all individual participants were collected, and patients’ data were anonymized. This study was conducted following the Declaration of Helsinki and has been written following the REMARK criteria [58].

The studied variables were age, tumor size, histological grade (Scarff–Bloom–Richardson classification modified by Elston and Ellis), vascular invasion, necrosis, nodal status, and immunophenotype. Survival data were obtained from clinical reports. Overall survival (OS) was defined as the period between surgery and patient’s death, and disease-free survival (DFS) as the time between surgery and recurrence. 

Patients were treated either with breast-conserving surgery or mastectomy. Post-lumpectomy radiotherapy was given at a total dose of 50 Gy after surgery, and those patients with HR-positive tumors were additionally treated with tamoxifen/aromatase inhibitors for 2–5 years. High-risk patients (young age, high histological grade, HR-negative tumors, or positive lymph nodes) received systemic chemotherapy after surgery, with 6 cycles of cyclophosphamide, methotrexate, 5-fluorouracil, or four cycles of doxorubicin plus cyclophosphamide. In our institution, trastuzumab (Herceptin^TM^) was approved to be added to chemotherapy protocols in HER2-positive BC patients after 2005. 

### 4.3. Immunohistochemistry (IHC) and FISH

Tissue microarrays (TMAs), including two cores of 1 mm from each formalin-fixed paraffin-embedded (FFPE) tumor, were made (Beecher Instruments) and stained with immunohistochemical standard techniques at conditions detailed in Supplementary Materials (Table S4). Two independent pathologists analyzed the results. Positive scores for staining were set as follows: HR (ER and PR) >1% (nuclei), Bcl2 > 50% (cytoplasm), p53 > 20% (nuclei), Ki67 ≥ 14% (nuclei), any degree of CK5/6 (cytoplasmic) or EGFR (membrane) [59] and HER2 according to the 2018 scoring guidelines (>10%, 3+cells). HER2 equivocal cases (2+ and <10% 3+) were confirmed by FISH (HER2 IQFISH pharmaDx^TM^; Agilent Technologies/Dako; Carpinteria, CA, USA) [60,61]. According to the St Gallen recommendation, intrinsic subtypes were classified based on the IHC results [62].

### 4.4. RNA Isolation and cDNA Synthesis

Two punches (1 mm thick/≥4 mm deep) of FFPE tissue from preselected areas were used for RNA isolation. Samples were first deparaffinized by grinding them into small pieces and incubating them with mineral oil (Sigma-Aldrich, Steinheim, Germany) at high temperature (95 °C). RNA isolation was carried out in the robotic workstation QIAcube (QIAGEN, Hilden, Germany) following the instructions of the RNeasy FFPE Kit (QIAGEN, Hilden, Germany) for tissue samples and RNeasy Minikit (QIAGEN, Hilden, Germany) for cell culture samples. A step of incubation with DNase (QIAGEN, Hilden, Germany) was included to eliminate any genomic DNA contamination. The final RNA concentration and purity were measured with a NanoDrop spectrophotometer (Thermo Scientific, Waltham MA, USA). For the reverse transcription reaction, 1.5 µg of RNA was used as a template in a total volume of 20 µL according to recommendations of the High-Capacity cDNA Reverse Transcription Kit (Thermo Fischer Scientific, Waltham MA, USA). 

### 4.5. Quantitative Real-Time PCR (qRT-PCR)

The qRT-PCR was carried out in a Real-Time PCR System 7500-FAST (Applied Biosystems, Foster City, CA, USA) using the TaqMan^®^ Fast Universal PCR Master Mix and TaqMan^®^ Gene Expression Assays (Life Technologies, CA, USA) containing specific exon-junction probes: *PUM1* (Hs00472881_m1), *β*-*ACTIN* (Hs01060665_g1), *ID1* (Hs03676575_s1), *ID2* (Hs04187239_m1), *ID3* (Hs00171409_m1) and *ID4* (Hs02912975_g1). Since *ID1* contains only one exon, we performed control reactions to ensure that the selected assay does not amplify genomic DNA. The qRT-PCR reaction took place in a total volume of 10 µl according to the manufacturers’ instructions. *PUM1* and *β*-*ACTIN* were used as reference genes. All reactions were done in duplicates, and no-template controls were included in each plate. Relative changes in mRNA expression were calculated as the fold change (FC) by the 2^-ΔΔCt^ method using as reference sample a pool of breast epithelium tissues from 10 healthy patients who underwent a breast reduction in the case of tissue samples, or the cell line 184A1 for BC cell lines. Tumor samples showing double expression than the control (FC ≥ 2) were considered overexpressed. Results were analyzed with the 7500 Software v2.06 (Applied Biosystems, Foster City, CA, USA). 

### 4.6. Statistical Analyses

The Kolmogorov–Smirnov test was used to identify whether variables were normally distributed. Parametric variables were defined by the average ± standard deviation, non-parametric variables were by the median and 25–75th percentiles or mean and range (min-max), and qualitative variables were defined by the frequency percentage of each subgroup. The Chi-square test was used to measure associations between qualitative variables. Log-rank tests were used for the comparison of Kaplan–Meier survival curves. Univariate and multivariate survival analyses were performed using the Cox proportional hazard model. All molecular and clinicopathological variables having a P-value < 0.05 in the univariate analyses were considered for the multivariate analyses. For the purpose of the study, samples were classified according to the expression FC as normal-low expression (FC < 2) versus overexpression (FC ≥ 2). 

In cell culture experiments, expression was defined as the average and standard deviation among replicates. Differences in expression among cell lines were compared with the Student’s T-test. *p*-values < 0.05 were regarded as statistically significant, whereas those between 0.05–0.10 were considered marginally significant. All statistical analyses were conducted using SPSS version 22.0 (SPSS Inc, Chicago, IL, USA).

## 5. Conclusions

*ID4*, which is the ID family member that differs the most in terms of sequence and function, is highly expressed in breast cancer cell lines than breast healthy tissue cell line. In breast cancer biopsies, *ID4* and *ID1* are overexpressed in a subset of samples that predominantly presented more aggressive phenotypes and shorter overall and disease-free survival. These results have been previously suggested for *ID1,* but there was not enough evidence showing a pro-oncogenic role of *ID4* in breast cancer.

## Figures and Tables

**Table 1:** Correlation between *ID1* and *ID4* mRNA expression and clinicopathological variables.

	Patients (*n* = 297)	*ID1*				*ID4*			
		*FC* < 2 (236)	FC ≥ 2 (61)	OR (95% CI)	*p* *	*FC* < 2 (248)	*FC* ≥ 2 (49)	OR (95% CI)	*p* *
		% (*n*)	% (*n*)			% (*n*)	% (*n*)		
**Age**									
≥50	188	64.4% (152)	59.0% (36)	0.8 (0.5–1.4)	ns	61.3% (152)	73.5% (36)	1.8 (0.9–3.5)	ns (0.071)
<50	109	35.6% (84)	41.0% (25)	1		38.7% (96)	26.5% (13)	1	
**Size**									
≥20	162	51.3% (121)	68.3% (41)	2.1 (1.1–3.7)	0.012	52.2% (129)	67.3% (33)	1.9 (1.0–3.6)	0.036
<20	134	48.7% (115)	31.7% (19)	1		47.8% (118)	32.7% (16)	1	
NA	1								
**Histological Grade**									
1	30	11.9% (28)	3.3% (2)	1		11.7% (29)	2.0% (1)	1	
2	89	33.1% (78)	18.0% (11)	2.0 (0.4–9.5)	ns	32.3% (80)	18.4% (9)	3.3 (0.4–26.9)	ns
3	178	55.1% (130)	78.7% (48)	5.2 (1.2–22.5)	0.029	56.0% (139)	79.6% (39)	8.1 (1.1-61.6)	0.042
**Necrosis**									
Present	100	29.9% (70)	50.0% (30)	2.3 (1.3–4.2)	0.003	30.5% (75)	52.1% (25)	2.5 (1.3–4.6)	0.004
Absent	194	70.1% (164)	50.0% (30)	1		69.5% (171)	47.9% (23)	1	
NA	3								
**Vascular Invasion**									
Present	107	33.8% (79)	45.9% (28)	1.7 (0.9–3.0)	ns (0.055)	33.3% (82)	51.0% (25)	2.1 (1.1–3.9)	0.015
Absent	188	66.2% (155)	54.1% (33)	1		66.7% (164)	49.0% (24)	1	
NA	2								
**Lymph Nodes Status**									
Positive	117	38.1% (90)	44.3% (27)	1.3 (0.7–2.3)	ns	37.9% (94)	46.9% (23)	1.5 (0.8–2.7)	ns
Negative	180	61.9% (146)	55.7% (34)	1		62.1% (154)	53.1% (26)	1	

OR: Odds Ratio; CI: Confidence Interval; NA: not available; ns: not significant (*p* > 0.05). * Chi-Square Test.

**Table 2:** Univariate and multivariate analysis of OS and DFS for all variables included in the study.

	Overall Survival				Disease-Free Survival			
	Univariate Analysis		Multivariate Analysis		Univariate Analysis		Multivariate Analysis	
	HR (95% CI)	*p* *	HR (95% CI)	*p* *	HR (95% CI)	*p* *	HR (95% CI)	*p* *
**Age**								
≥50	1.3 (0.7–2.3)	ns			0.7 (0.4–1.1)	ns		
<50	1				1			
**Size**								
≥20	2.8 (1.5–5.2)	0.002	1.7 (0.8–3.5)	ns	1.8 (1.0–3.2)	0.034	1.3 (0.7–2.4)	ns
<20	1		1		1			
**Histological Grade**								
1	1		1		1			
2	4.2 (0.5–32.9)	ns	2.5 (0.3–20.0)	ns	1.3 (0.4–3.9)	0.702		
3	8.1 (1.1–58.8)	0.040	2.6 (0.3–21.4)	ns	2.1 (0.8–5.9)	0.157		
**Necrosis**								
Present	1.8 (1.0–3.2)	0.042	1.4 (0.7–2.6)	ns	1.8 (1.0–3.1)	0.041	1.8 (1.0–3.1)	0.050
Absent	1		1		1			
**Vascular Invasion**								
Present	2.6 (1.4–4.6)	0.001	1.6 (0.8–3.1)	ns	2.5 (1.4–4.2)	0.001	1.9 (1.1–3.5)	0.031
Absent	1		1		1			
**Lymph Nodes**								
Positive	2.6 (1.4–4.6)	0.001	1.6 (0.8–3.1)	ns	1.6 (0.9–2.7)	0.107		
Negative	1		1		1			
**HR Status**								
Negative	1.8 (1.0–3.3)	0.039	1.3 (0.6–2.4)	ns	1.6 (0.9–2.8)	ns (0.080)		
Positive	1		1		1			
**ID1 FC**								
≥2	1.3 (0.7–2.5)	ns			2.0 (1.1–3.6)	0.016	0.6 (0.3–1.5)	ns
<2	1				1			
**ID2 FC**								
≥2	0.8 (0.4–1.7)	ns			0.9 (0.4–1.7)	ns		
<2	1				1			
**ID3 FC**								
≥2	1.0 (0.5–2.3)	ns			0.9 (0.4–1.9)	ns		
<2	1				1			
**ID4 FC**								
≥2	2.1 (1.2–3.9)	0.016	1.6 (0.8–3.0)	ns (0.167)	3.4 (1.9–6.0)	<0.001	4.0 (1.8–8.9)	0.001
<2	1		1		1			

HR, hazard ratio; CI, confidence interval; HR status, hormonal receptor status; ns, not significant (*p* > 0.05). * Cox model.

## Data Availability

The data presented in this study are available on request from the corresponding author.

## Supplementary Materials

The following are available online at https://www.mdpi.com/2072-6694/13/3/492/s1, Figure S1: Percentage of samples overexpressing ID2 and ID3 in each BC immunophenotype, Figure S2: Kaplan-Meier plot for OS and DFS rates (%) for all patients classified according to breast cancer immunophenotypes, Figure S3: Kaplan-Meier plot for OS and DFS rates (%) for patients with HER2-enriched tumors classified according to ID1 or ID4 mRNA expression. Table S1: Correlation between ID2 and ID3 mRNA expression and clinic-pathological variables, Table S2: Correlations between BC immunophenotypes and OS, Table S3: Correlations between BC immunophenotypes and DFS, Table S4: Antibodies and conditions for immunohistochemistry.

## References

[1] Ferlay J., Colombet M., Soerjomataram I., Mathers C., Parkin D.M., Pineros M., Znaor A., Bray F. (2019). Estimating the global cancer incidence and mortality in 2018: GLOBOCAN sources and methods. Int. J. Cancer.

[2] Niell B.L., Freer P.E., Weinfurtner R.J., Arleo E.K., Drukteinis J.S. (2017). Screening for Breast Cancer. Radiol. Clin. N. Am..

[3] Coates A.S., Winer E.P., Goldhirsch A., Gelber R.D., Gnant M., Piccart-Gebhart M., Thurlimann B., Senn H.J., Panel M. (2015). Tailoring therapies--improving the management of early breast cancer: St Gallen International Expert Consensus on the Primary Therapy of Early Breast Cancer 2015. Ann. Oncol..

[4] SEER Cancer Statistics Review, 1975–2015. https://seer.cancer.gov/csr/1975_2015/.

[5] Matsumoto A., Jinno H., Ando T., Fujii T., Nakamura T., Saito J., Takahashi M., Hayashida T., Kitagawa Y. (2016). Biological markers of invasive breast cancer. Jpn. J. Clin. Oncol..

[6] American Cancer Society. https://www.cancer.org/cancer/breast-cancer/understanding-a-breast-cancer-diagnosis/breast-cancer-survival-rates.html.

[7] Stovgaard E.S., Nielsen D., Hogdall E., Balslev E. (2018). Triple negative breast cancer - prognostic role of immune-related factors: A systematic review. Acta Oncol..

[8] Benezra R., Davis R.L., Lockshon D., Turner D.L., Weintraub H. (1990). The protein Id: A negative regulator of helix-loop-helix DNA binding proteins. Cell.

[9] Ling F., Kang B., Sun X.H. (2014). Id proteins: Small molecules, mighty regulators. Curr. Top. Dev. Biol..

[10] Riechmann V., van Cruchten I., Sablitzky F. (1994). The expression pattern of Id4, a novel dominant negative helix-loop-helix protein, is distinct from Id1, Id2 and Id3. Nucleic Acids Res..

[11] Roschger C., Cabrele C. (2017). The Id-protein family in developmental and cancer-associated pathways. Cell Commun. Signal..

[12] Zhang N., Yantiss R.K., Nam H.S., Chin Y., Zhou X.K., Scherl E.J., Bosworth B.P., Subbaramaiah K., Dannenberg A.J., Benezra R. (2014). ID1 is a functional marker for intestinal stem and progenitor cells required for normal response to injury. Stem Cell Rep..

[13] Colacino J.A., Azizi E., Brooks M.D., Harouaka R., Fouladdel S., McDermott S.P., Lee M., Hill D., Madden J., Boerner J. (2018). Heterogeneity of Human Breast Stem and Progenitor Cells as Revealed by Transcriptional Profiling. Stem Cell Rep..

[14] Nam H.S., Benezra R. (2009). High levels of Id1 expression define B1 type adult neural stem cells. Cell Stem Cell..

[15] Schaefer B.M., Koch J., Wirzbach A., Kramer M.D. (2001). Expression of the helix-loop-helix protein ID1 in keratinocytes is upregulated by loss of cell-matrix contact. Exp. Cell Res..

[16] Lasorella A., Benezra R., Iavarone A. (2014). The ID proteins: Master regulators of cancer stem cells and tumour aggressiveness. Nat. Rev. Cancer.

[17] Amaral L.H.P., Bufalo N.E., Peres K.C., Barreto I.S., Campos A., Ward L.S. (2019). ID Proteins May Reduce Aggressiveness of Thyroid Tumors. Endocr Pathol..

[18] Damdinsuren B., Nagano H., Kondo M., Yamamoto H., Hiraoka N., Yamamoto T., Marubashi S., Miyamoto A., Umeshita K., Dono K. (2005). Expression of Id proteins in human hepatocellular carcinoma: Relevance to tumor dedifferentiation. Int. J. Oncol..

[19] Ding R., Han S., Lu Y., Guo C., Xie H., Zhang N., Song Z., Cai L., Liu J., Dou K. (2010). Overexpressed Id-1 is associated with patient prognosis and HBx expression in hepatitis B virus-related hepatocellular carcinoma. Cancer Biol. Ther..

[20] Ciarrocchi A., Piana S., Valcavi R., Gardini G., Casali B. (2011). Inhibitor of DNA binding-1 induces mesenchymal features and promotes invasiveness in thyroid tumour cells. Eur. J. Cancer.

[21] Lin C.Q., Singh J., Murata K., Itahana Y., Parrinello S., Liang S.H., Gillett C.E., Campisi J., Desprez P.Y. (2000). A role for Id-1 in the aggressive phenotype and steroid hormone response of human breast cancer cells. Cancer Res..

[22] Perk J., Gil-Bazo I., Chin Y., de Candia P., Chen J.J., Zhao Y., Chao S., Cheong W., Ke Y., Al-Ahmadie H. (2006). Reassessment of id1 protein expression in human mammary, prostate, and bladder cancers using a monospecific rabbit monoclonal anti-id1 antibody. Cancer Res..

[23] Schoppmann S.F., Schindl M., Bayer G., Aumayr K., Dienes J., Horvat R., Rudas M., Gnant M., Jakesz R., Birner P. (2003). Overexpression of Id-1 is associated with poor clinical outcome in node negative breast cancer. Int. J. Cancer.

[24] Wazir U., Jiang W.G., Sharma A.K., Newbold R.F., Mokbel K. (2013). The mRNA expression of inhibitors of DNA binding-1 and -2 is associated with advanced tumour stage and adverse clinical outcome in human breast cancer. Anticancer Res..

[25] Zhou X.L., Zeng D.E., Ye Y.H., Sun S.M., Lu X.F., Liang W.Q., Chen C.F., Lin H.Y. (2018). Prognostic values of the inhibitor of DNAbinding family members in breast cancer. Oncol. Rep..

[26] Li K., Yao L., Chen L., Cao Z.G., Yu S.J., Kuang X.Y., Hu X., Shao Z.M. (2014). ID2 predicts poor prognosis in breast cancer, especially in triple-negative breast cancer, and inhibits E-cadherin expression. Onco. Targets Ther..

[27] Zhou J.D., Ma J.C., Zhang T.J., Li X.X., Zhang W., Wu D.H., Wen X.M., Xu Z.J., Lin J., Qian J. (2017). High bone marrow ID2 expression predicts poor chemotherapy response and prognosis in acute myeloid leukemia. Oncotarget.

[28] Wieczorek A., Balwierz W. (2015). The Role of Id2 Protein in Neuroblatoma in Children. Pathol. Oncol. Res..

[29] Stighall M., Manetopoulos C., Axelson H., Landberg G. (2005). High ID2 protein expression correlates with a favourable prognosis in patients with primary breast cancer and reduces cellular invasiveness of breast cancer cells. Int. J. Cancer.

[30] Huang L., Cai J., Guo H., Gu J., Tong Y., Qiu B., Wang C., Li M., Xia L., Zhang J. (2019). ID3 Promotes Stem Cell Features and Predicts Chemotherapeutic Response of Intrahepatic Cholangiocarcinoma. Hepatology.

[31] Sachindra L.L., Novak D., Wu H., Huser L., Granados K., Orouji E., Utikal J. (2017). New role of ID3 in melanoma adaptive drug-resistance. Oncotarget.

[32] Chen Y.S., Aubee J., DiVito K.A., Zhou H., Zhang W., Chou F.P., Simbulan-Rosenthal C.M., Rosenthal D.S. (2015). Id3 induces an Elk-1-caspase-8-dependent apoptotic pathway in squamous carcinoma cells. Cancer Med..

[33] Chen F.F., Lv X., Zhao Q.F., Xu Y.Z., Song S.S., Yu W., Li X.J. (2018). Inhibitor of DNA binding 3 reverses cisplatin resistance in human lung adenocarcinoma cells by regulating the PI3K/Akt pathway. Oncol. Lett..

[34] Patel D., Morton D.J., Carey J., Havrda M.C., Chaudhary J. (2015). Inhibitor of differentiation 4 (ID4): From development to cancer. Biochim. Biophys. Acta.

[35] Jen Y., Manova K., Benezra R. (1996). Expression patterns of Id1, Id2, and Id3 are highly related but distinct from that of Id4 during mouse embryogenesis. Dev. Dyn..

[36] Sharma P., Chinaranagari S., Chaudhary J. (2015). Inhibitor of differentiation 4 (ID4) acts as an inhibitor of ID-1, -2 and -3 and promotes basic helix loop helix (bHLH) E47 DNA binding and transcriptional activity. Biochimie.

[37] Zhang Y., Zhang L.X., Liu X.Q., Zhao F.Y., Ge C., Chen T.Y., Yao M., Li J.J. (2017). Id4 promotes cell proliferation in hepatocellular carcinoma. Chin. J. Cancer.

[38] Zeng W., Rushing E.J., Hartmann D.P., Azumi N. (2010). Increased inhibitor of differentiation 4 (id4) expression in glioblastoma: A tissue microarray study. J. Cancer.

[39] Peretz Y., Wu H., Patel S., Bellacosa A., Katz R.A. (2015). Inhibitor of DNA Binding 4 (ID4) is highly expressed in human melanoma tissues and may function to restrict normal differentiation of melanoma cells. PLoS ONE.

[40] Sharma P., Chinaranagari S., Patel D., Carey J., Chaudhary J. (2012). Epigenetic inactivation of inhibitor of differentiation 4 (Id4) correlates with prostate cancer. Cancer Med..

[41] Noetzel E., Veeck J., Niederacher D., Galm O., Horn F., Hartmann A., Knuchel R., Dahl E. (2008). Promoter methylation-associated loss of ID4 expression is a marker of tumour recurrence in human breast cancer. BMC Cancer.

[42] Beger C., Pierce L.N., Kruger M., Marcusson E.G., Robbins J.M., Welcsh P., Welch P.J., Welte K., King M.C., Barber J.R. (2001). Identification of Id4 as a regulator of BRCA1 expression by using a ribozyme-library-based inverse genomics approach. Proc. Natl. Acad. Sci. USA.

[43] Welcsh P.L., Lee M.K., Gonzalez-Hernandez R.M., Black D.J., Mahadevappa M., Swisher E.M., Warrington J.A., King M.C. (2002). BRCA1 transcriptionally regulates genes involved in breast tumorigenesis. Proc. Natl. Acad. Sci. USA.

[44] Cheang M.C., Chia S.K., Voduc D., Gao D., Leung S., Snider J., Watson M., Davies S., Bernard P.S., Parker J.S. (2009). Ki67 index, HER2 status, and prognosis of patients with luminal B breast cancer. J. Natl. Cancer Inst..

[45] Kaplan-Meier Plotter. https://kmplot.com/analysis/.

[46] Jang K.S., Han H.X., Paik S.S., Brown P.H., Kong G. (2006). Id-1 overexpression in invasive ductal carcinoma cells is significantly associated with intratumoral microvessel density in ER-negative/node-positive breast cancer. Cancer Lett..

[47] de Candia P., Akram M., Benezra R., Brogi E. (2006). Id4 messenger RNA and estrogen receptor expression: Inverse correlation in human normal breast epithelium and carcinoma. Hum. Pathol..

[48] Turner N.C., Reis-Filho J.S., Russell A.M., Springall R.J., Ryder K., Steele D., Savage K., Gillett C.E., Schmitt F.C., Ashworth A. (2007). BRCA1 dysfunction in sporadic basal-like breast cancer. Oncogene.

[49] Agboola A.O.J., Banjo A.A.F., Anunobi C., Salami B., Deji-Agboola M., Musa A., Nolan C.C., Rakha E.A., Green Ellis I.O.A.R. (2014). Helix-loop-helix protein inhibitor of differentiation 4 (ID4) expression is an indicator of poor survival in Nigerian breast cancer women. J. Afr. Cancer.

[50] Thike A.A., Tan P.H., Ikeda M., Iqbal J. (2016). Increased ID4 expression, accompanied by mutant p53 accumulation and loss of BRCA1/2 proteins in triple-negative breast cancer, adversely affects survival. Histopathology.

[51] Donzelli S., Milano E., Pruszko M., Sacconi A., Masciarelli S., Iosue I., Melucci E., Gallo E., Terrenato I., Mottolese M. (2018). Expression of ID4 protein in breast cancer cells induces reprogramming of tumour-associated macrophages. Breast Cancer Res..

[52] Fontemaggi G., Dell’Orso S., Trisciuoglio D., Shay T., Melucci E., Fazi F., Terrenato I., Mottolese M., Muti P., Domany E. (2009). The execution of the transcriptional axis mutant p53, E2F1 and ID4 promotes tumor neo-angiogenesis. Nat. Struct. Mol. Biol..

[53] Junankar S., Baker L.A., Roden D.L., Nair R., Elsworth B., Gallego-Ortega D., Lacaze P., Cazet A., Nikolic I., Teo W.S. (2015). ID4 controls mammary stem cells and marks breast cancers with a stem cell-like phenotype. Nat. Commun..

[54] Baker L.A., Holliday H., Roden D., Krisp C., Wu S.Z., Junankar S., Serandour A.A., Mohammed H., Nair R., Sankaranarayanan G. (2020). Proteogenomic analysis of Inhibitor of Differentiation 4 (ID4) in basal-like breast cancer. Breast Cancer Res..

[55] Ruzinova M.B., Benezra R. (2003). Id proteins in development, cell cycle and cancer. Trends Cell Biol..

[56] Olmeda D., Moreno-Bueno G., Flores J.M., Fabra A., Portillo F., Cano A. (2007). SNAI1 is required for tumor growth and lymph node metastasis of human breast carcinoma MDA-MB-231 cells. Cancer Res..

[57] Wojnarowicz P.M., Lima E.S.R., Ohnaka M., Lee S.B., Chin Y., Kulukian A., Chang S.H., Desai B., Garcia Escolano M., Shah R. (2019). A Small-Molecule Pan-Id Antagonist Inhibits Pathologic Ocular Neovascularization. Cell Rep..

[58] McShane L.M., Altman D.G., Sauerbrei W., Taube S.E., Gion M., Clark G.M. (2006). REporting recommendations for tumor MARKer prognostic studies (REMARK). Breast Cancer Res. Treat..

[59] Peiro G., Adrover E., Sanchez-Tejada L., Lerma E., Planelles M., Sanchez-Paya J., Aranda F.I., Giner D., Gutierrez-Avino F.J. (2011). Increased insulin-like growth factor-1 receptor mRNA expression predicts poor survival in immunophenotypes of early breast carcinoma. Mod. Pathol..

[60] Sanmartin E., Ortiz-Martinez F., Pomares-Navarro E., Garcia-Martinez A., Rodrigo-Banos M., Garcia-Escolano M., Andres L., Lerma E., Aranda F.I., Martinez-Peinado P. (2017). CD44 induces FOXP3 expression and is related with favorable outcome in breast carcinoma. Virchows Arch..

[61] Wolff A.C., Hammond M.E.H., Allison K.H., Harvey B.E., Mangu P.B., Bartlett J.M.S., Bilous M., Ellis I.O., Fitzgibbons P., Hanna W. (2018). Human Epidermal Growth Factor Receptor 2 Testing in Breast Cancer: American Society of Clinical Oncology/College of American Pathologists Clinical Practice Guideline Focused Update. J. Clin. Oncol..

[62] Maisonneuve P., Disalvatore D., Rotmensz N., Curigliano G., Colleoni M., Dellapasqua S., Pruneri G., Mastropasqua M.G., Luini A., Bassi F. (2014). Proposed new clinicopathological surrogate definitions of luminal A and luminal B (HER2-negative) intrinsic breast cancer subtypes. Breast Cancer Res..

