# Supplementary material for: ID1 and ID4 Are Biomarkers of Tumor Aggressiveness and Poor Outcome in Immunophenotypes of Breast Cancer
Source: Cancers (Basel). 2021 Jan 27;13(3):492. doi: 10.3390/cancers13030492 (PMC7865969; doi:10.3390/cancers13030492)
Supplement: Supplementary file 1 [file cancers-13-00492-s001.pdf]

Article

ID1 and ID4 are Biomarkers of Tumor Aggressiveness and Poor Outcome in Immunophenotypes of Breast Cancer

Marta Garcia-Escolano, Yoel G. Montoyo-Pujol, Fernando Ortiz-Martinez, Jose J. Ponce, Silvia Delgado-Garcia, Tina A. Martin, Hortensia Ballester, F. Ignacio Aranda, Elena Castellon-Molla, J. Miguel Sempere-Ortells and Gloria Peiro

Table S1. Correlation between *ID2* and *ID3* mRNA expression and clinical-pathological variables.

	Patients (n = 297)	*ID2* <2 (214) % (n)	*ID2* ≥2 (83) % (n)	OR (CI95)	*p* *	*ID3* <2 (236) % (n)	*ID3* ≥2 (61) % (n)	OR (CI95)	*p* *
**Age**									
≥50	188	62.1% (133)	66.3% (55)	1.2 (0.7–2.0)	ns	63.9% (146)	68.9% (42)	1.4 (0.8–2.5)	ns
<50	109	37.9% (81)	33.7% (28)	1		38.1% (90)	31.1% (19)	1	
**Size**									
≥20	162	55.4% (118)	53.0% (44)	0.9 (0.6–1.5)	ns	54.9% (129)	54.1% (33)	1.0 (0.6–1.7)	ns
<20	134	44.6% (95)	47.0% (39)	1		54.1% (106)	45.9% (28)	1	
NA	1					ns		ns	
**Histological Grade**									
1	30	10.3% (22)	9.6% (8)	1		10.6% (25)	8.2% (5)	1	
2	89	31.3% (67)	26.5% (22)	0.9 (0.4–2.3)	ns	30.1% (71)	29.5% (18)	1.3 (0.4–3.8)	ns
3	178	58.4% (125)	63.9% (53)	1.2 (0.5–2.8)	ns	59.3% (140)	62.3% (38)	1.4 (0.5–3.8)	ns
**Necrosis**									
Present	100	36.5% (77)	27.7% (23)	0.7 (0.4–1.2)	ns (0.097)	35.5% (83)	28.3% (17)	0.7 (0.4–1.3)	ns
Absent	194	63.5% (134)	72.3% (60)	1		64.5% (151)	71.7% (43)	1	
NA	3								
**Vascular Invasion**									
Present	107	38.2% (81)	31.3% (26)	0.7 (0.4–1.3)	ns	36.6% (86)	35.0% (21)	0.9 (0.5–1.7)	ns
Absent	188	61.8% (131)	68.7% (57)	1		63.4% (149)	65.0% (39)	1	
NA	2								

**Lymph Nodes**									
Positive	117	40.2% (86)	37.3% (31)	0.9 (0.5–1.5)	ns	37.7% (89)	45.9% (28)	1.4 (0.8–2.5)	ns
Negative	180	59.8% (128)	62.7% (52)	1		62.3% (147)	54.1% (33)	1	

NA: not available; ns: not significant (p>0.05).* Chi Square Test.

Table S2. Correlations between clinical-pathological variables and OS (Kaplan-Meier method; log-rank test).

	**Patients (n)**	**Survival %**	***p* ***
**Phenotype**			
Luminal A-like	55	90.2	
Luminal B/HER2-	54	91.5	0.027
Luminal B/HER2+	40	83.3	
HER2-enriched	27	65.9	
TNBL	82	83.7	

* Log-rank test.

Table S3. Correlations between clinical-pathological variables and DFS (Kaplan-Meier method; log-rank test).

	**Patients (n)**	**Survival %**	***p* ***
**Phenotype**			
Luminal A-like	54	88.5	
Luminal B/HER2-	52	88.1	
Luminal B/HER2+	38	79.2	<0.001
HER2-enriched	24	58.5	
TNBL	85	86.7	

* Log-rank test.

Table S4. Antibodies and conditions for immunohistochemistry.

**Antibody**	**Vendor**	**Clone**	**Dilution**
ER	Dako/Agilent	ER-α	1:50
PR	Dako/Agilent	PgR636	1:50
HER2	Dak/Agilent o	HercepTest™	NA
p53	Dako/Agilent	DO7	1:50
Ki67	Dako/Agilent	MIB-I	1:100

Bcl2	Neomarkers	Bcl2/100/D5	1:50
CK5/6	Dako/Agilent	D5/16B4	1:100
EGFR	Dako/Agilent	Pharmaco Dx™	NA

NA: Not available. Dako/Agilent Technol (Santa Clara, CA); NeoMarkers Inc (Fremont, CA, USA).

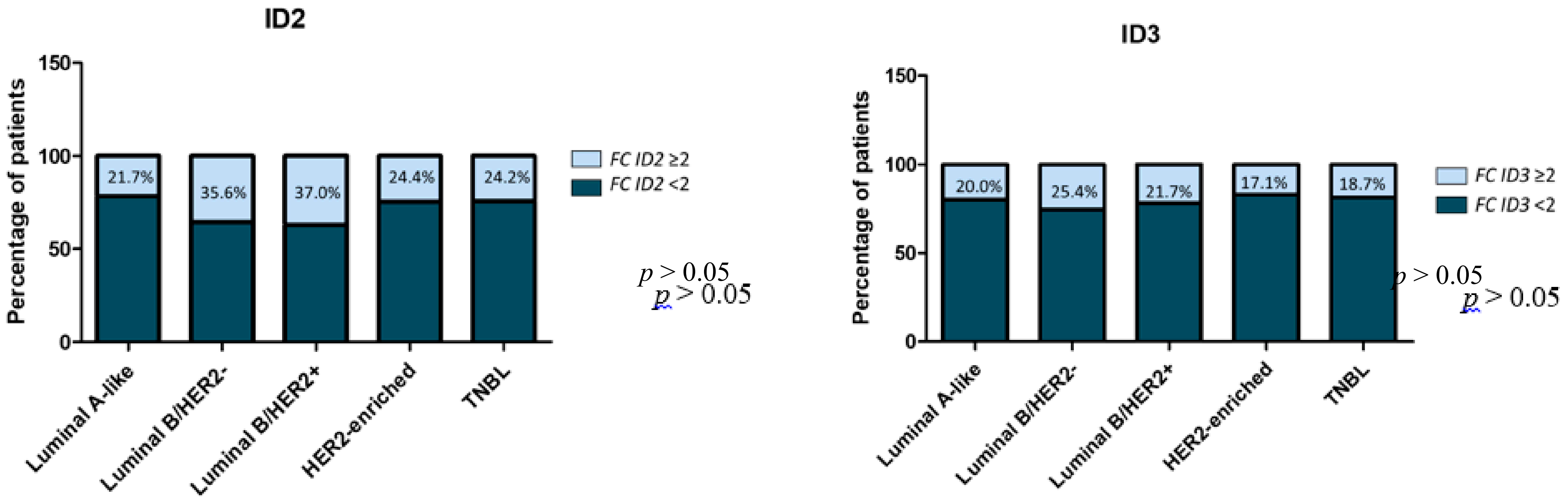

Figure S1. Percentage of samples overexpressing ID2 (**A**) and ID3 (**B**) in each BC immunophenotype.

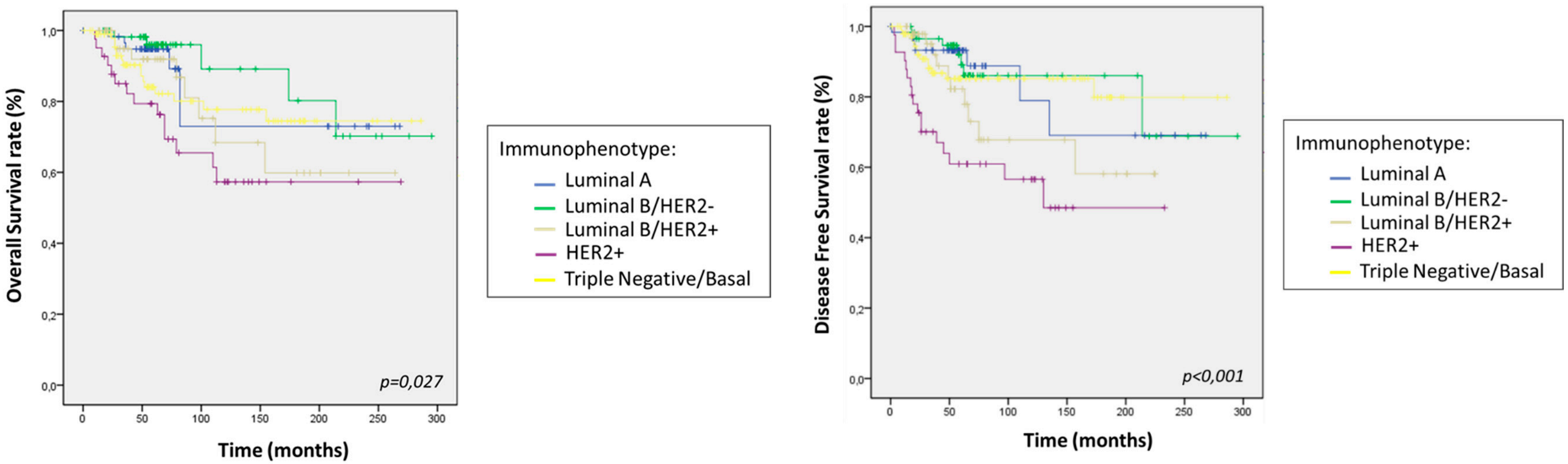

A **B**

Figure S2. Kaplan-Meier plot for OS (**A**) and DFS (**B**) rates (%) for all patients classified according to breast cancer immunophenotypes.

A

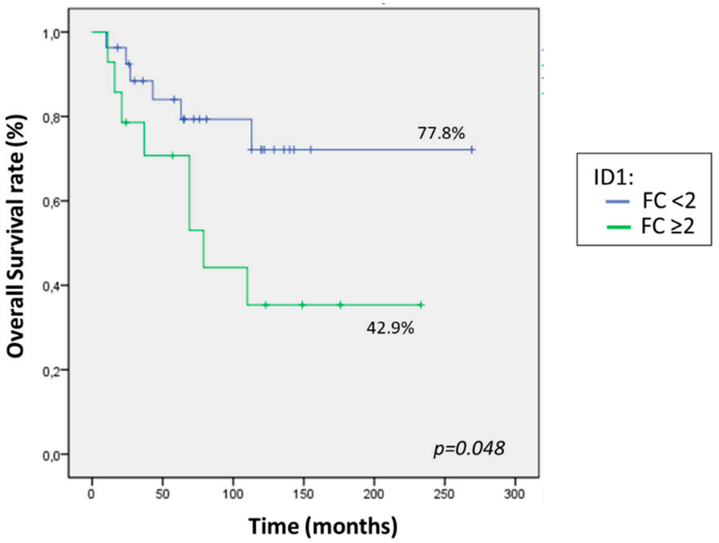
Overall Survival rate (%)
1,0
0,8
0,6
0,4
0,2
0,0
0
50
100
150
200
250
300
Time (months)
77.8%
42.9%
p=0.048
ID1:
FC <2
FC ≥2

B

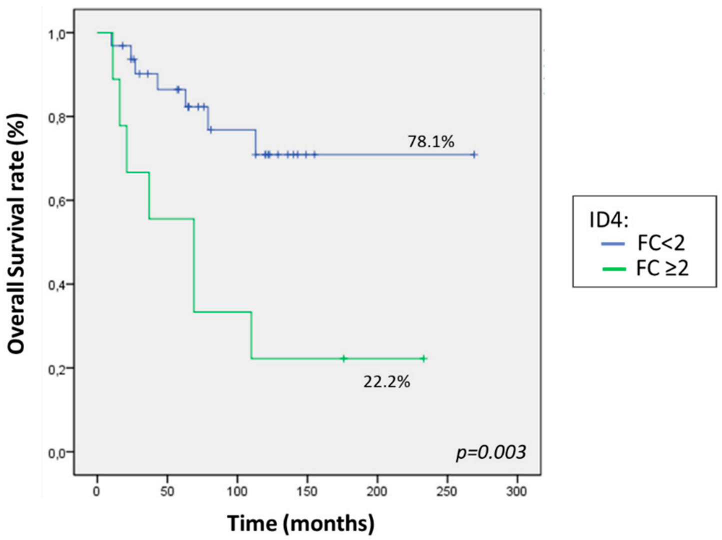
Overall Survival rate (%)
1,0
0,8
0,6
0,4
0,2
0,0
0
50
100
150
200
250
300
Time (months)
78.1%
22.2%
p=0.003
ID4:
FC<2
FC ≥2

C

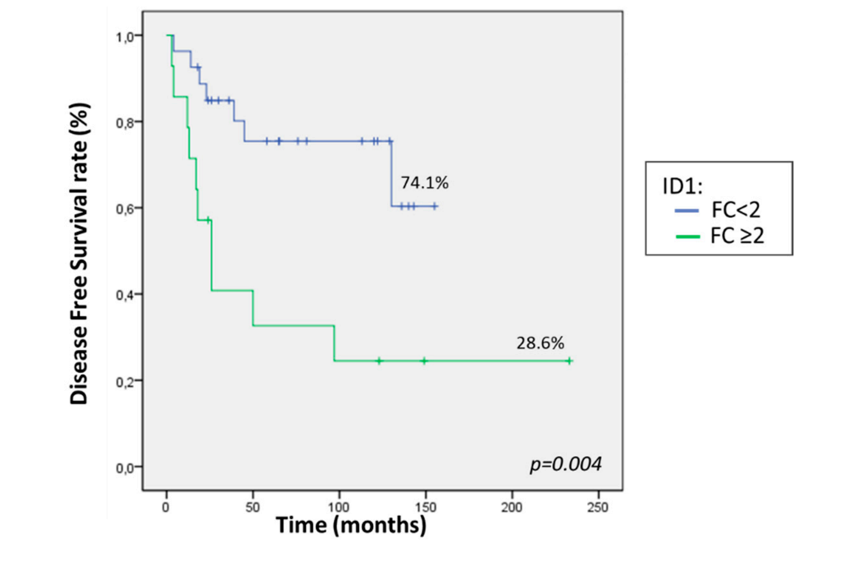

D

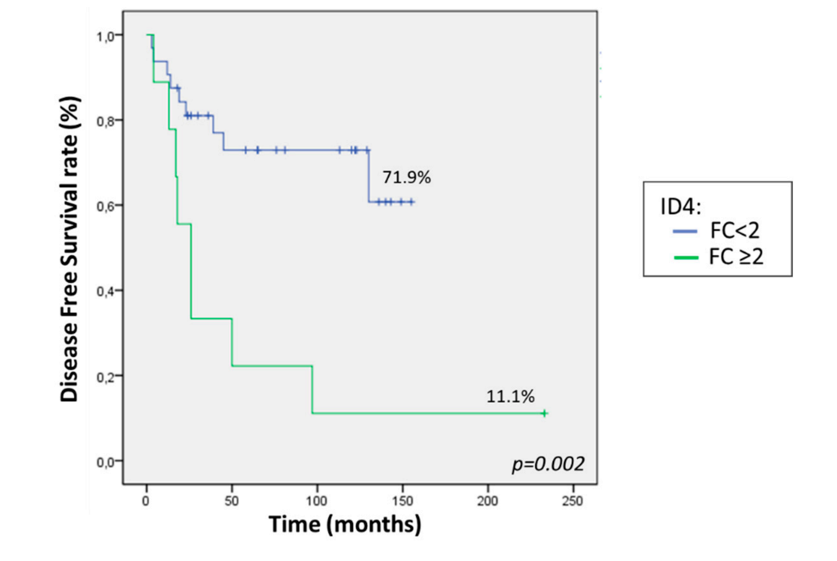

Figure S3. Kaplan-Meier plot for OS (**A**,**B**) and DFS (**C**,**D**) rates (%) for patients with HER2-enriched tumors classified according to ID1 (**A**,**C**) or ID4 (**B**,**D**) mRNA expression.
